# Global, regional, and national burden of blindness and vision loss due to common eye diseases along with its attributable risk factors from 1990 to 2019: a systematic analysis from the global burden of disease study 2019

**DOI:** 10.18632/aging.203374

**Published:** 2021-08-09

**Authors:** Xiaorong Yang, Hui Chen, Tongchao Zhang, Xiaolin Yin, Jinyu Man, Qiufeng He, Ming Lu

**Affiliations:** 1 Clinical Epidemiology Unit, Qilu Hospital of Shandong University, Jinan, China; 2 Clinical Research Center of Shandong University, Qilu Hospital, Cheeloo College of Medicine, Shandong University, Jinan, China; 3 Department of Epidemiology and Health Statistics, School of Public Health, Cheeloo College of Medicine, Shandong University, Jinan, China

**Keywords:** blindness and vision loss, global burden, risk factors, temporal trends, prevention

## Abstract

To map the magnitudes and temporal trends of blindness and vision loss (BVL) due to common eye diseases along with its attributable risk factors at the national, regional, and global levels. The annual burden of BVL in 204 countries and territories was extracted from the Global Burden of Disease Study 2019. The estimated annual percentage change (EAPC) and causes composition change were calculated to quantify the temporal trends of BVL-related disease burden by sex, region, and eye disease. The global disability-adjusted life years (DALYs) of BVL increased from 12.44 million in 1990 to 22.56 million in 2019, with a slightly decreased rate from 3.03 to 2.78 per 1000 population (EAPC = -0.30). About 29.6% of BVL-related DALYs worldwide were caused by cataract, followed by refraction disorders (29.1%), near vision loss (21.7%), other vision loss (13.7%), glaucoma (3.3%), and age-related macular degeneration (2.5%) in 2019. The age-standardized DALYs rates due to each eye disease type in most regions were decreased, especially in countries with high burden and high-middle socio-demographic index. Moreover, the contribution of smoking and air pollution from solid fuels to BVL burden decreased, however, the age-standardized burden of BVL attributed to high body-mass index and high fasting plasma glucose elevated gradually across almost all regions. The temporal trend of BVL burden due to specific eye diseases varies remarkably by region, sex and age. Understanding the real-time patterns of BVL burden is crucial for formulating more effective and targeted prevention and healthcare strategies to decrease the BVL burden.

## INTRODUCTION

The latest global estimation and project on blindness and distance and near vision impairment among the population aged 50 years and older documents that about 43.3 million people worldwide suffer from blindness and 295 million people have moderate and severe vision impairment in 2020 [1]. Globally, an estimated 22.6 million disability-adjusted life years (DALYs) were caused by blindness and vision loss (BVL) in 2019, accounting for 0.88% of total DALYs due to all causes [2]. BVL brings severe personal educational and financial difficulties [3], reduces the quality of daily living [4], and increases mortality [5]. More than one-third of the patients with vision impairment could have not received effective prevention and treatment [6]. Understanding the global, regional and national characteristics as well as the temporal trends in specific causes of BVL is the essential basis for targeted public policy-making, such as medical resource allocation and health program planning.

The updated global and regional prevalence of BVL categorized by the severity of visual impairment along with the eye diseases has been assessed among the population aged over 50 years old [1, 7], which provides comprehensive knowledge for understanding the landscape of BVL burden. But the burden of the entire population and the impact of potential risk factors have not been assessed yet. The latest Global Burden of Disease (GBD) 2019 Study comprehensively estimated the annual burden of 369 diseases using extensive data sources and robust statistical methods in 204 countries and territories from 1990 to 2019 [2], which provided a unique opportunity to grasp the progress of the BVL burden. In this study, we aim to retrieve the global, regional, and national prevalence and DALYs of BVL caused by six major eye diseases by sex and age group from 1990 to 2019 based on the GBD 2019 Study, and further estimate the changes in the DALYs due to specific eye disease of BVL attributed to potential risk factors. Our results will serve as an important supplement and extension to previous studies [1, 7], optimize the process of universal eye health care, and facilitate the establishment of targeted eye health strategies tailoring to different characteristics in the future.

## RESULTS

### The global burden due to overall BVL

Globally, the accumulated number of BVL patients increased from 353.2 (95% uncertainty interval [UI]: 298.7, 414.0) million in 1990 to 713.9 (95% UI: 593.2, 841.1) million in 2019, with a stable age-standardized prevalence rate (ASPR) of about 8.5 cases per 100 people (Supplementary Table 1). The estimated DALYs of BVL worldwide increased from 12.44 (95% UI: 8.64, 17.38) million in 1990 to 22.56 (95% UI: 15.58, 31.74) million in 2019, while the estimated annual percentage change (EAPC) in age-standardized DALYs rate (-0.30; 95% confidence interval [CI]: -0.34, -0.25) showed a slight decline from 3.03 to 2.78 per 1000 population during the period (Table 1). The ASPR and age-standardized DALYs rate of BVL among women were somewhat greater than that among men (9.10/100 vs. 8.25/100 in 2019, 2.91/1000 vs. 2.64/1000 in 2019, respectively), and the gender disparity in BVL burden further widened gradually over the past 30 years (Table 1 and Supplementary Table 1).

**Table 1:** DALYs and age-standardized DALYs rate per 1000 people for all blindness and vision loss in 1990 and 2019, and its estimated annual percentage change from 1990 to 2019.

**Characteristics**	**1990**			**2019**			**1990-2019**
	**DALYs No.×10^5^ (95% UI)**	**Age-standardized DALYs rate per 1000 No. (95% UI)**		**DALYs No.×10^5^ (95% UI)**	**Age-standardized DALYs rate per 1000 No. (95% UI)**		**EAPC in age-standardized DALYs Rate No. (95% CI)**
**Overall**	124.4 (86.4, 173.8)	3.03 (2.12, 4.21)		225.6 (155.7, 317.4)	2.78 (1.92, 3.92)		-0.30 (-0.34, -0.25)*
**Sex**							
Males	56.4 (38.81, 79.01)	2.96 (2.08, 4.11)		100.7 (69.1, 142.4)	2.64 (1.82, 3.72)		-0.40 (-0.45, -0.36)*
Females	68.02 (47.26, 94.69)	3.11 (2.18, 4.31)		124.9 (86.4, 175.0)	2.91 (2.01, 4.09)		-0.22 (-0.26, -0.17)*
**SDI**							
High SDI	10.76 (7.42, 14.92)	1.13 (0.77, 1.56)		16.75 (11.59, 23.17)	1.08 (0.73, 1.52)		-0.16 (-0.20, -0.12)*
High-middle SDI	25.96 (17.85, 36.81)	2.48 (1.72, 3.51)		45.15 (30.91, 64.58)	2.35 (1.61, 3.40)		-0.17 (-0.22, -0.11)*
Middle SDI	41.33 (28.77, 57.19)	3.95 (2.79, 5.42)		80.07 (55.00, 112.7)	3.32 (2.29, 4.63)		-0.58 (-0.64, -0.52)*
Low-middle SDI	34.17 (23.87, 47.50)	5.53 (3.94, 7.57)		59.55 (41.07, 83.20)	4.35 (3.02, 6.05)		-0.82 (-0.86, -0.77)*
Low SDI	12.14 (8.39, 16.90)	4.91 (3.48, 6.72)		23.96 (16.49, 33.37)	4.35 (3.05, 5.98)		-0.42 (-0.48, -0.36)*
**GBD region**							
High-income Asia Pacific	1.94 (1.36, 2.66)	1.05 (0.74, 1.44)		3.35 (2.37, 4.56)	0.97 (0.67, 1.34)		-0.28 (-0.33, -0.23)*
High-income North America	2.82 (1.95, 3.91)	0.87 (0.59, 1.21)		4.36 (3.03, 6.02)	0.84 (0.58, 1.17)		-0.12 (-0.16, -0.08)*
Western Europe	6.90 (4.85, 9.37)	1.38 (0.97, 1.89)		9.28 (6.52, 12.58)	1.27 (0.88, 1.74)		-0.27 (-0.29, -0.24)*
Australasia	0.23 (0.16, 0.32)	1.08 (0.74, 1.51)		0.41 (0.29, 0.56)	1.02 (0.69, 1.42)		-0.17 (-0.21, -0.14)*
Tropical Latin America	3.73 (2.63, 5.09)	3.67 (2.60, 4.97)		7.18 (5.03, 9.76)	3.06 (2.14, 4.17)		-0.28 (-0.42, -0.14)*
Andean Latin America	0.92 (0.65, 1.26)	4.06 (2.90, 5.48)		1.82 (1.26, 2.54)	3.20 (2.23, 4.45)		-0.93 (-0.99, -0.88)*
Central Latin America	3.28 (2.28, 4.52)	3.50 (2.48, 4.78)		6.74 (4.65, 9.40)	2.85 (1.97, 3.95)		-0.69 (-0.73, -0.66)*
Southern Latin America	0.74 (0.52, 1.02)	1.61 (1.13, 2.20)		1.11 (0.77, 1.51)	1.43 (0.99, 1.97)		-0.36 (-0.38, -0.34)*
Caribbean	0.77 (0.53, 1.07)	2.82 (1.96, 3.90)		1.22 (0.84, 1.72)	2.39 (1.65, 3.38)		-0.56 (-0.58, -0.55)*
Eastern Europe	6.85 (4.63, 9.90)	2.61 (1.77, 3.76)		8.02 (5.3, 11.91)	2.50 (1.66, 3.69)		-0.26 (-0.30, -0.21)*
Central Europe	2.40 (1.58, 3.62)	1.72 (1.13, 2.56)		3.09 (1.99, 4.69)	1.58 (1.03, 2.40)		-0.29 (-0.30, -0.27)*
Central Asia	1.49 (1.01, 2.11)	3.12 (2.15, 4.40)		2.00 (1.36, 2.87)	2.76 (1.88, 3.95)		-0.46 (-0.48, -0.43)*
North Africa and Middle East	7.84 (5.56, 10.77)	4.15 (2.96, 5.57)		13.93 (9.74, 19.11)	3.15 (2.22, 4.28)		-0.95 (-0.96, -0.94)*
South Asia	38.94 (26.86, 54.07)	6.71 (4.78, 9.15)		72.99 (50.55, 101.9)	5.18 (3.62, 7.19)		-0.90 (-0.95, -0.85)*
Southeast Asia	13.71 (9.73, 18.64)	5.30 (3.82, 7.11)		23.31 (16.44, 31.85)	4.00 (2.85, 5.43)		-1.02 (-1.07, -0.98)*
East Asia	22.63 (15.43, 32.70)	2.63 (1.81, 3.77)		48.09 (32.41, 71.39)	2.43 (1.65, 3.56)		-0.19 (-0.36, -0.03)*
Oceania	0.11 (0.08, 0.16)	3.59 (2.50, 4.93)		0.24 (0.17, 0.34)	3.33 (2.31, 4.65)		-0.26 (-0.34, -0.18)*
Western Sub-Saharan Africa	3.89 (2.74, 5.41)	4.27 (3.01, 5.86)		8.48 (5.86, 11.93)	4.18 (2.91, 5.78)		-0.18 (-0.28, -0.08)*
Eastern Sub-Saharan Africa	3.42 (2.38, 4.75)	4.32 (3.03, 5.90)		6.55 (4.54, 9.16)	3.73 (2.62, 5.13)		-0.51 (-0.54, -0.48)*
Central Sub-Saharan Africa	0.56 (0.37, 0.85)	2.35 (1.57, 3.46)		1.25 (0.81, 1.90)	2.19 (1.44, 3.28)		-0.20 (-0.27, -0.14)*
Southern Sub-Saharan Africa	1.24 (0.84, 1.80)	4.23 (2.92, 6.09)		2.14 (1.42, 3.25)	3.66 (2.46, 5.48)		-0.57 (-0.64, -0.50)*
**Specific eye diseases**							
Glaucoma	4.42 (3.02, 6.26)	0.13 (0.09, 0.18)		7.48 (5.16, 10.45)	0.10 (0.07, 0.13)		-1.00 (-1.04, -0.95)*
Cataract	34.93 (24.82, 47.2)	0.93 (0.66, 1.25)		66.76 (47.61, 90.06)	0.83 (0.59, 1.12)		-0.23 (-0.31, -0.14)*
Age-related macular degeneration	2.97 (2.05, 4.19)	0.08 (0.06, 0.12)		5.64 (3.93, 7.89)	0.07 (0.05, 0.10)		-0.71 (-0.78, -0.65)*
Refraction disorders	40.81 (27.56, 57.87)	0.89 (0.61, 1.24)		65.69 (44.34, 92.91)	0.82 (0.55, 1.15)		-0.31 (-0.35, -0.27)*
Near vision loss	22.56 (10.35, 44.41)	0.56 (0.26, 1.09)		49.01 (22.56, 96.69)	0.59 (0.27, 1.17)		0.05 (0.00, 0.11)
Other vision loss	18.73 (13.37, 25.56)	0.45 (0.32, 0.61)		31.00 (22.06, 42.43)	0.38 (0.27, 0.52)		-0.65 (-0.70, -0.60)*

DALYs, disability-adjusted life years; No., number; UI, uncertainty interval; EAPC, estimated annual percentage change; CI, confidential interval; SDI, socio-demographic index; GBD, global burden of disease.

* *P* value<0.05.

### Variation in BVL burden at the national and regional level

**Figure 1:**
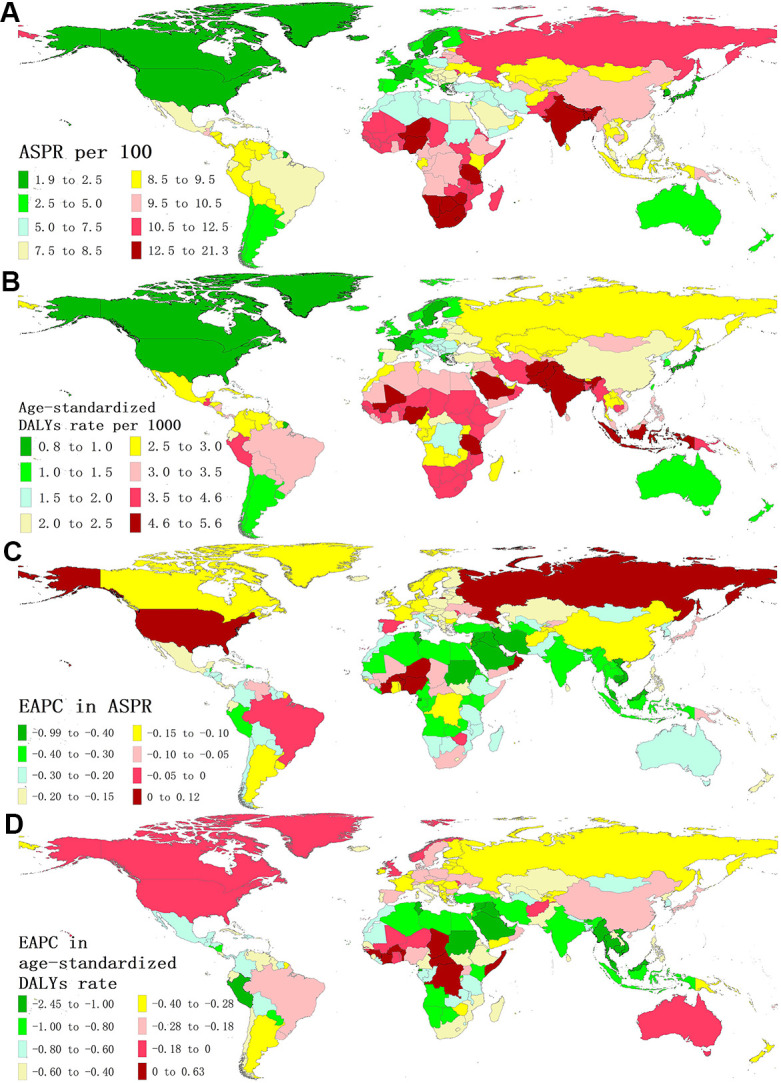
**The global disease burden of BVL for both sexes in 204 countries and territories.** (**A**) The ASPR of BVL in 2019; (**B**) The age-standardized DALYs rate of BVL in 2019; (**C**) The EAPC in ASPR of BVL from 1990 to 2019. (**D**) The EAPC in age-standardized DALYs rate of BVL from 1990 to 2019. BVL, blindness and vision loss; ASPR, age-standardized prevalence rate; DALYs, disability-adjusted life years; EAPC, estimated annual percentage change.

The global variety of ASPR and age-standardized DALYs rate of BVL were around 11 and 7 times, respectively, in 2019 (Figure 1), with the highest ASPR in Nepal (21.27/100) and age-standardized DALYs rate in Indonesia (5.59/1000), and the lowest ASPR in Sweden (1.94/100) and age-standardized DALYs rate in Canada (0.81/1000). Overall, the ASPR in 2019 was higher than 11/100 in 29 countries and territories, including Tanzania, Zimbabwe, Lesotho, India, etc., (Figure 1A and Supplementary Table 2), which also showed a severe burden in age-standardized DALYs rate (Figure 1B and Supplementary Table 3). Conversely, Japan, Canada, Greenland and other 8 countries presented an ASPR of less than 2.5/100 (Figure 1A). In Equatorial Guinea, both ASPR and age-standardized DALYs rate had the largest decline (EAPC = -0.99; EAPC = -2.45, respectively) (Figure 1C, 1D). The top declines in age-standardized DALYs rate were also observed in Saudi Arabia, Cambodia, Qatar and Tunisia (Supplementary Table 4). Only 6 countries and territories (Burkina Faso, Cote d’Ivoire, Benin, Central African Republic, Chad, and Somalia) reported a significantly increasing age-standardized DALYs rate from 1990 and 2019 (Figure 1D and Supplementary Table 5).

For five socio-demographic index (SDI) regions, the number of DALYs of BVL increased across all SDI regions (Table 1 and Supplementary Figure 1), while the corresponding age-standardized DALYs rates decreased (Table 1). The greatest decrease observed in low-middle SDI region (EAPC = -0.82; 95% CI: -0.86, -0.77). As for GBD regions, absolute numbers in DALYs of BVL also increased in all regions (Table 1), but all 21 GBD regions showed a significant reduction in age-standardized DALYs rate of BVL, with the largest reduction found in Southeast Asia (EAPC = -1.02; 95% CI: -1.07, -0.98).

### BVL burden due to specific eye diseases

**Figure 2:**
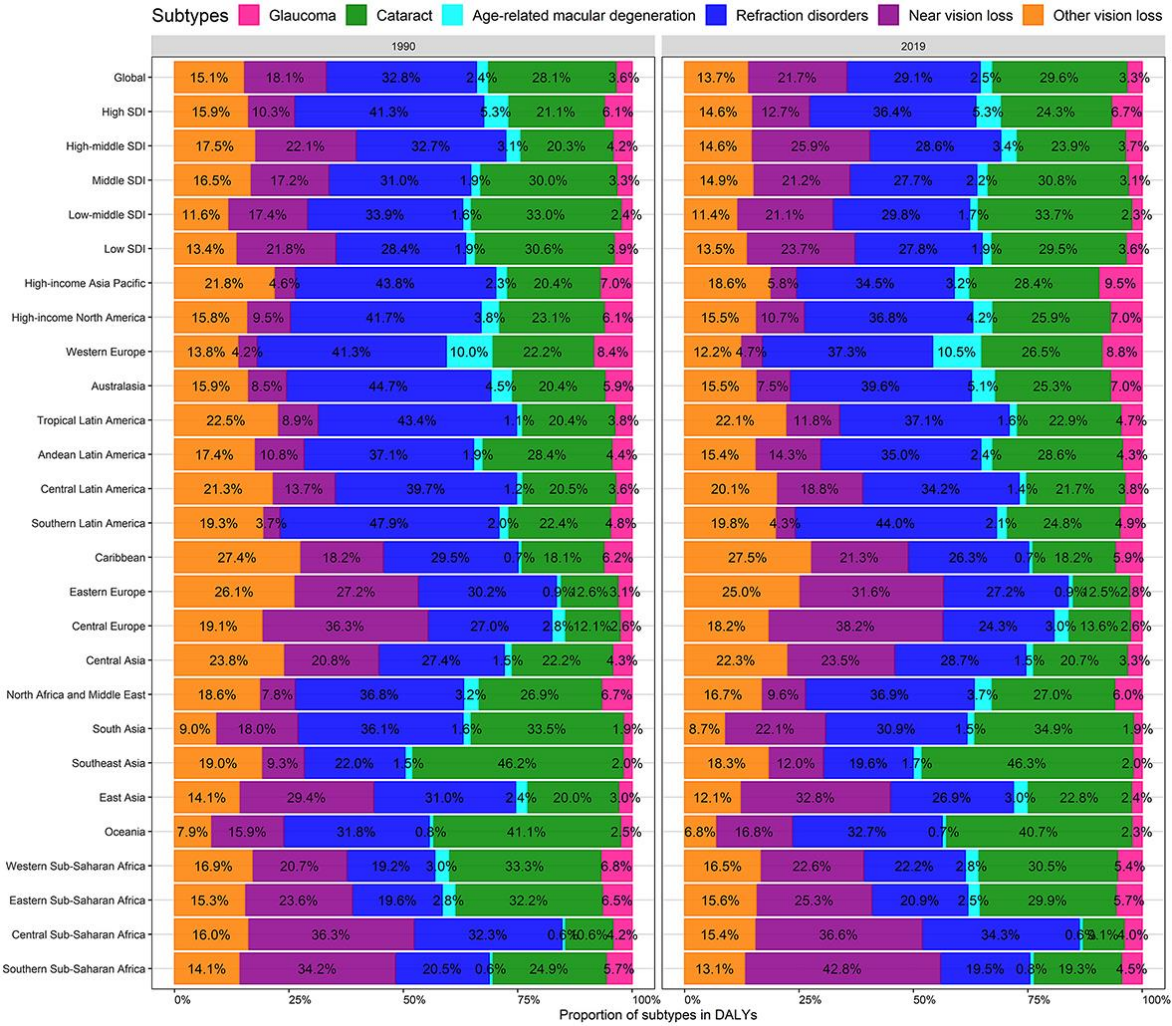
**Contribution of glaucoma, cataract, age-related macular degeneration, refraction disorders, near vision loss, and, other vision loss to absolute DALYs of BVL, both sexes, globally and by region, in 1990 and 2019.** DALYs, disability-adjusted life years; BVL, blindness and vision loss.

The distribution of BVL-related DALYs due to specific eye diseases at the global and regional levels between 1990 and 2019 are presented in Supplementary Figure 1 and Figure 2. The BVL-related DALYs number due to all six eye diseases at global and regional levels increased during the monitoring period, especially for the near vision loss (Table 1 and Supplementary Figure 1). Globally, about 29.6% of BVL-related DALYs were caused by cataract, followed by refraction disorders (29.1%), near vision loss (21.7%), other vision loss (13.7%), glaucoma (3.3%) and age-related macular degeneration (2.5%) in 2019 (Figure 2). Compared with the proportions in 1990, the decrease in refraction disorders (-3.7%) and the increase in near vision loss (3.6%) in 2019 were notable. Besides, the proportions presented a great temporal and spatial variation. For instance, in Southern Latin America, the proportion of refraction disorders dropped from 47.9% in 1990 to 44.0% in 2019, and the proportion of age-related macular degeneration in West Europe (10.5% in 2019) was much higher than that in other regions.

The age-standardized BVL burden rate along with the temporal trend due to specific eye diseases was significantly heterogeneous across the world (Supplementary Figures 2, 3). Overall, the age-standardized DALYs rates due to each categorized eye disease in most regions and countries were decreased. However, the age-standardized DALYs rate due to age-related macular degeneration increased in some countries located in Sub-Saharan Africa; the near vision loss burden increased in Eastern Europe; and the refraction disorders burden increased in Western Sub-Saharan Africa, Tropical Latin America, and Australasia.

### The BVL burden and age structure

**Figure 3:**
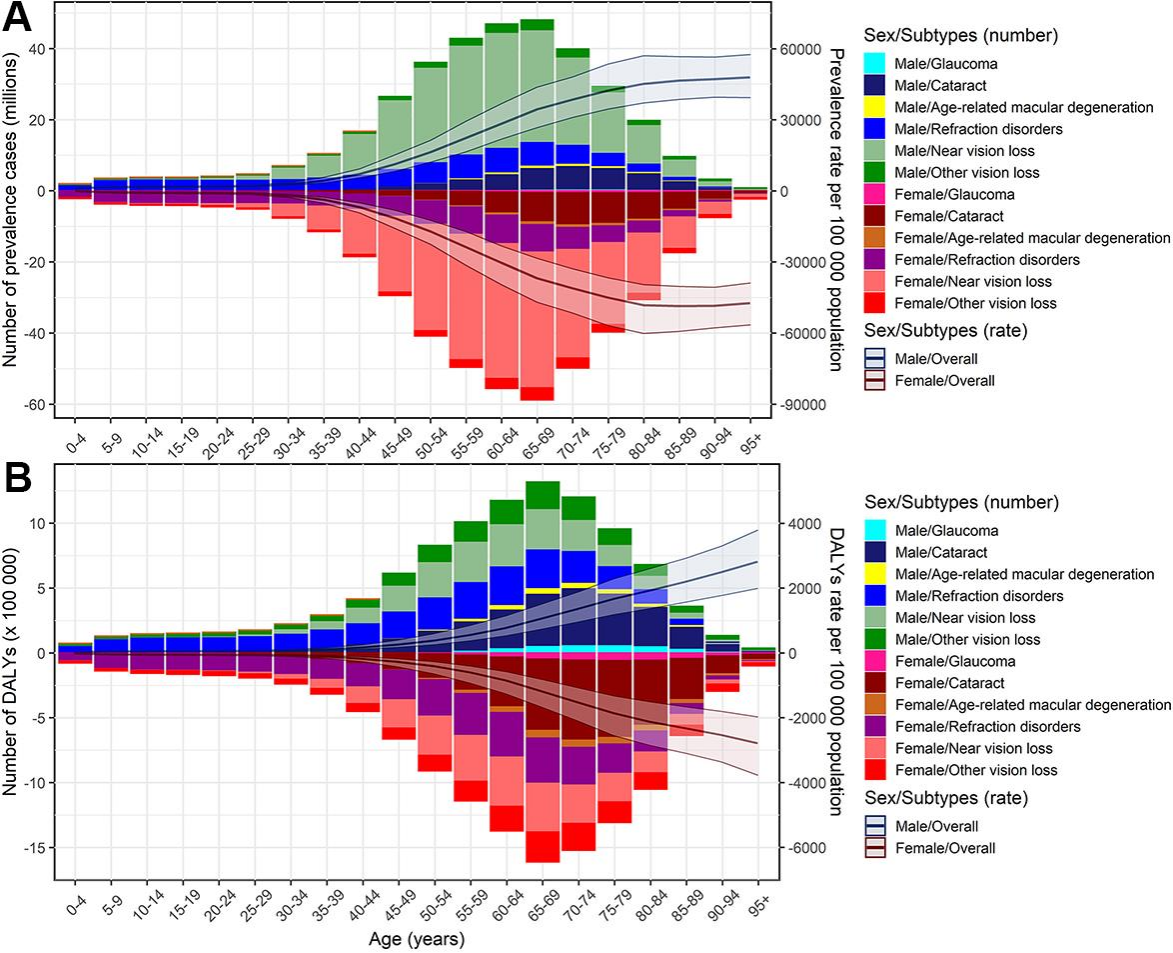
**Age-specific counts and rates of BVL burden by different eye diseases, by sex, 2019.** (**A**) prevalence; (**B**) DALYs. BVL, blindness and vision loss; DALYs, disability-adjusted life years.

**Figure 4:**
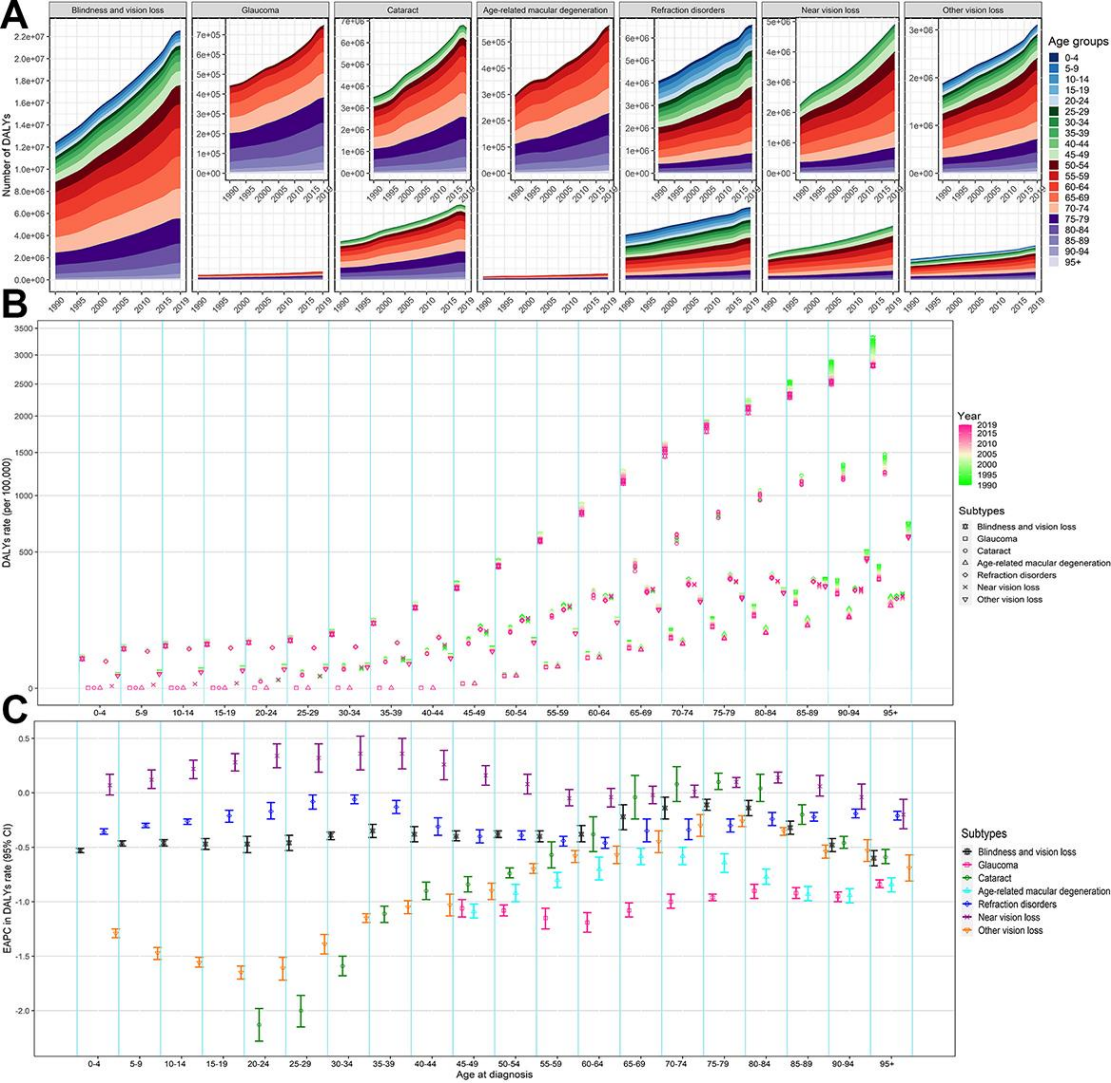
**The change of DALYs of BVL by different eye diseases and age groups, both sexes, from 1990 to 2019.** (**A**) Number of DALYs; (**B**) DALYs rate; (**C**) EAPC in DALYs rate. The figures of six eye diseases are further enlarged in its top-right panel. BVL, blindness and vision loss; DALYs, disability-adjusted life years; EAPC, estimated annual percentage change.

Figure 3 presents the age distribution of BVL-related burden by specific eye diseases in 2019. The near vision loss was the predominant subtype in the prevalence of BVL across almost all age groups for both sexes. The proportion of refraction disorders in DALYs became pronounced across all age groups and the cataract was changed to an overwhelming subtype for DALYs due to BVL among the elders aged 60 years and above. The burden rate of overall BVL kept pace with age increasing, especially after 55 years of age. The proportion of patients over 50 years old steadily increased among the DALYs number due to BVL for all six eye diseases (Figure 4A). Although the EAPCs in DALYs due to total BVL among all age groups were closed to -0.5, the remarkable dropped rate of burden due to cataract and other vision loss could be observed among the people under 50 years old (Figure 4B, 4C). Furthermore, the DALYs rate due to near vision loss among aged 5-49 years presented a slight upward trend.

### The influential factors for EAPC

**Figure 5:**
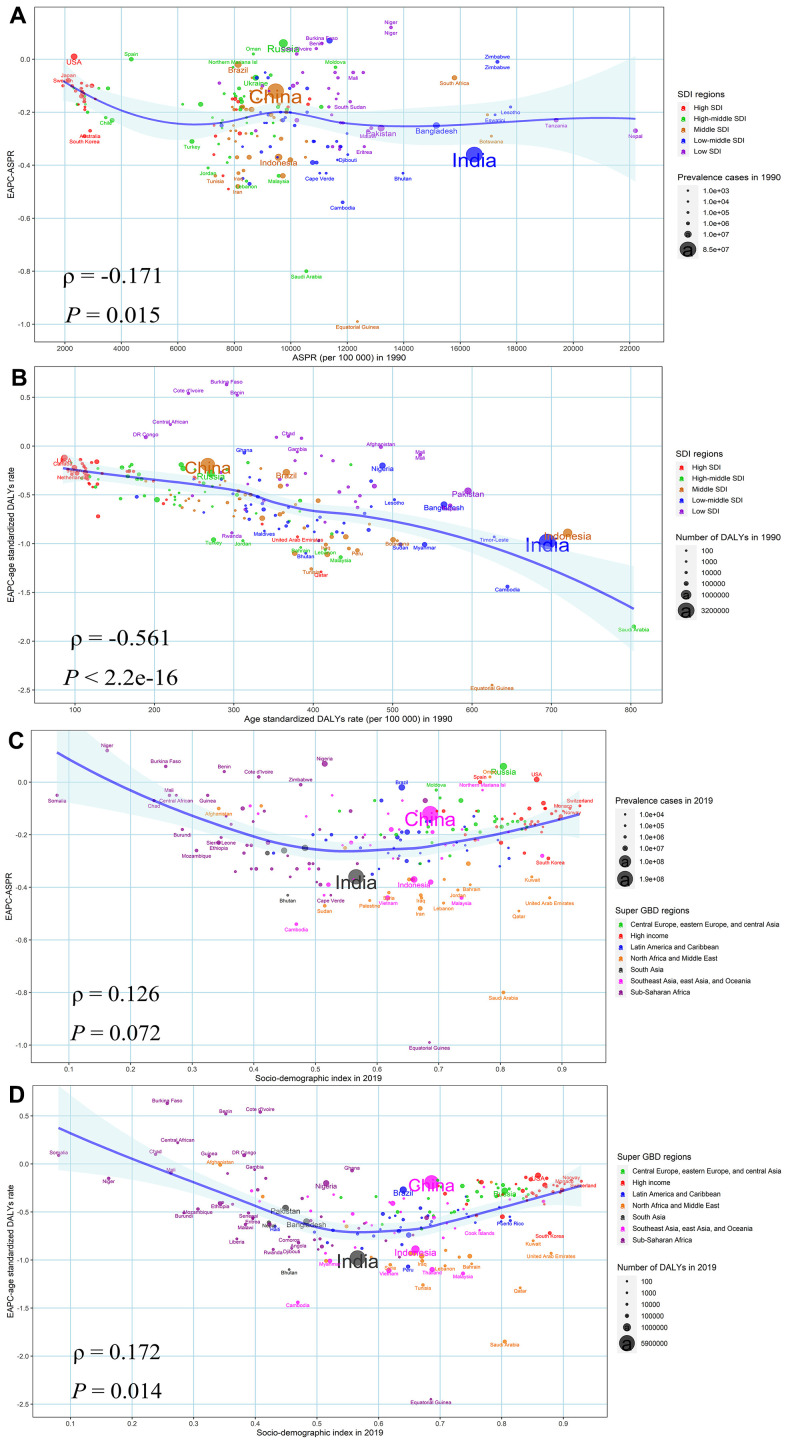
**The factors affected the EAPCs in age-standardized burden rate of BVL from 1990 to 2019, both sexes, at the national level.** (**A**) ASPR of BVL in 1990 and EAPC in ASPR (**B**) SDI in 2019 and EAPC in ASPR; (**C**) age-standardized DALYs rate of BVL in 1990 and EAPC in age-standardized DALYs rate; (**D**) SDI in 2019 and EAPC in age-standardized DALYs rate. The circles represent countries and the size of circle is increased with the number of DALYs. The ρ indices and *P* values presented were derived from Spearman rank analysis. The blue line and its shade was fitted by LOESS. ASPR, age-standardized prevalence rate; EAPC, estimated annual percentage change; DALYs, disability-adjusted life years; BVL, blindness and vision loss; SDI, socio-demographic index.

**Figure 6:**
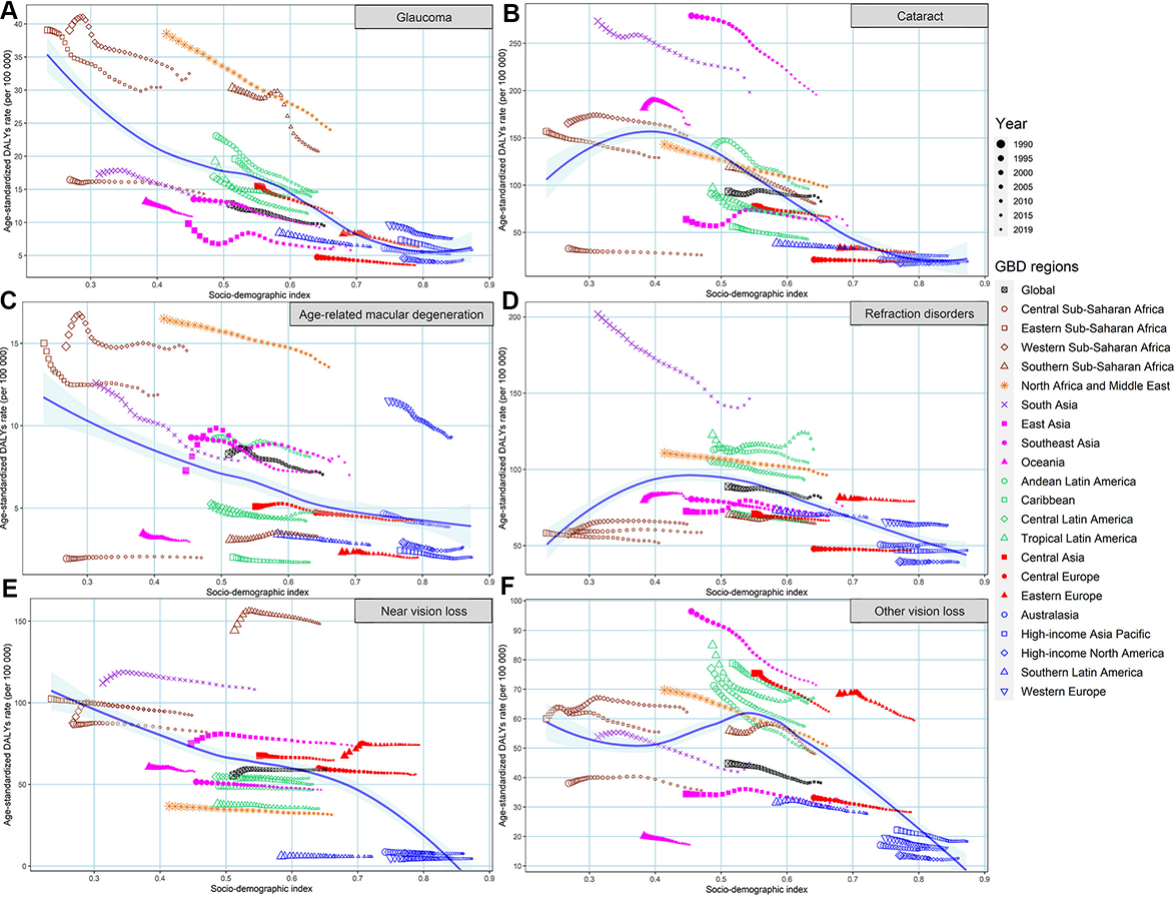
**The changing trend in age-standardized DALYs rates of BVL across 21 GBD regions with SDI, by eye diseases, both sexes, from 1990 to 2019.** (**A**) glaucoma; (**B**) cataract; (**C**) age-related macular degeneration; (**D**) refraction disorders; (**E**) near vision loss; (**F**) other vision loss. DALYs, disability-adjusted life years; BVL, blindness and vision loss; GBD, global burden of disease; SDI, socio-demographic index.

As shown in Figure 5, a significant negative association was detected between EAPC and age-standardized overall BVL burden rates in 1990 at the national level (ρ = -0.171, *P* = 0.015 for prevalence; ρ = -0.561, *P* < 2.2e-16 for DALYs), which reflected that the BVL burden might be given priority intervention in countries with a high burden rate. Although the positive relationship between EAPC in age-standardized burden rate and SDI in 2019 was significant, the obvious U shape relationship could be observed. Moreover, we investigated the correlation between SDI and age-standardized DALYs rate by specific eye diseases from 1990 to 2019 in 21 GBD regions around the world (Supplementary Figure 4 and Figure 6). The results indicated that the age-standardized DALYs rate was markedly negatively associated with SDI in GBD regions with SDI ranging from 0.4 to 0.7, especially for the burden in glaucoma, cataract, age-related macular degeneration and other vision loss.

### The BVL-related DALYs attributable to risk factors

**Figure 7:**
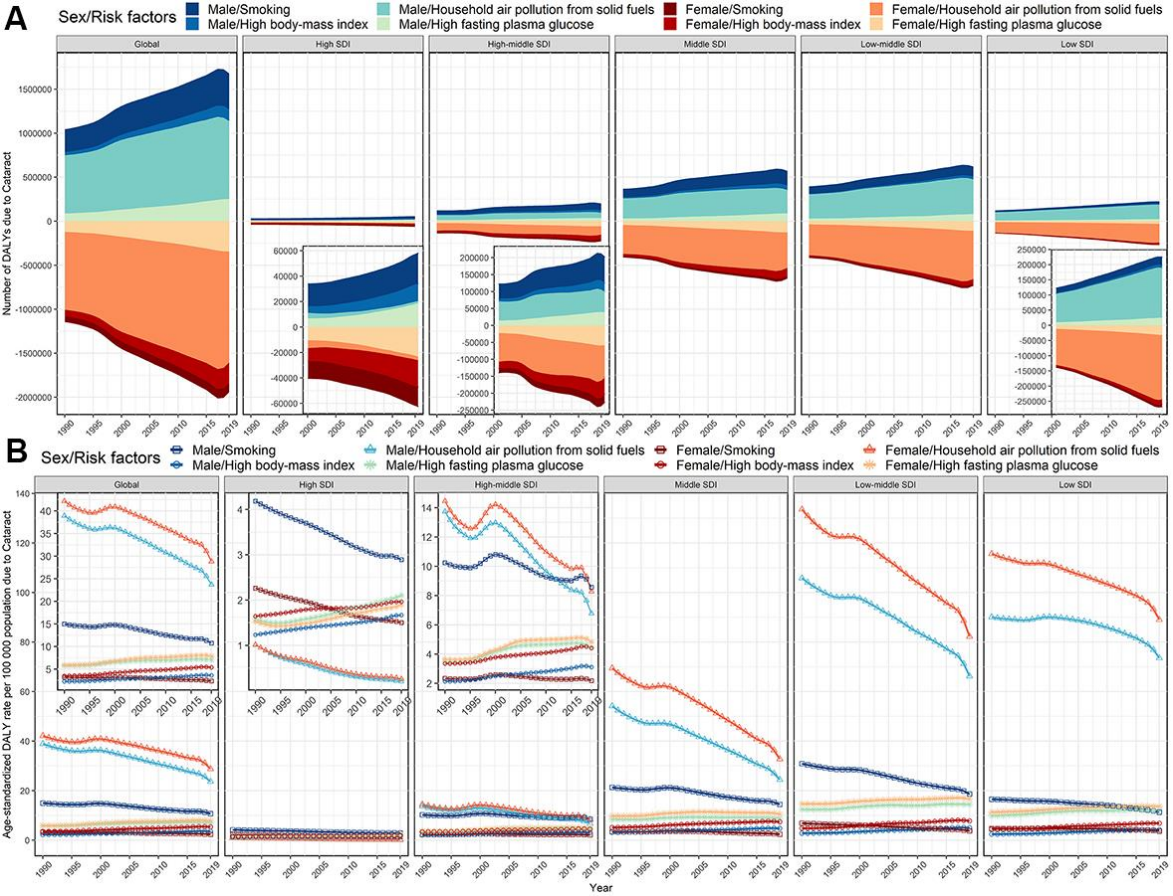
**Risk factors contributing to cataract-related DALYs, globally and by SDI regions, both sexes, from 1990 to 2019.** (**A**) number of DALYs; (**B**) age-standardized DALYs rate. DALYs, disability-adjusted life years; SDI, socio-demographic index.

We searched the identified potential risk factors for specific BVL disorders from the GBD database. Eventually, we found four risk factors involved in cataract: smoking, high body-mass index, household air pollution from solid fuels, and high fasting plasma glucose. Of these risk factors, household air pollution from solid fuels remained the greatest contributor to cataract-related DALYs, particularly for females in lower SDI regions, although the corresponding age-standardized DALYs rate gradually decreased from 1990 to 2019 (Figure 7). Moreover, we could observe the incremental influence on cataract from the high body-mass index and high fasting plasma glucose across almost all SDI regions.

**Figure 8:**
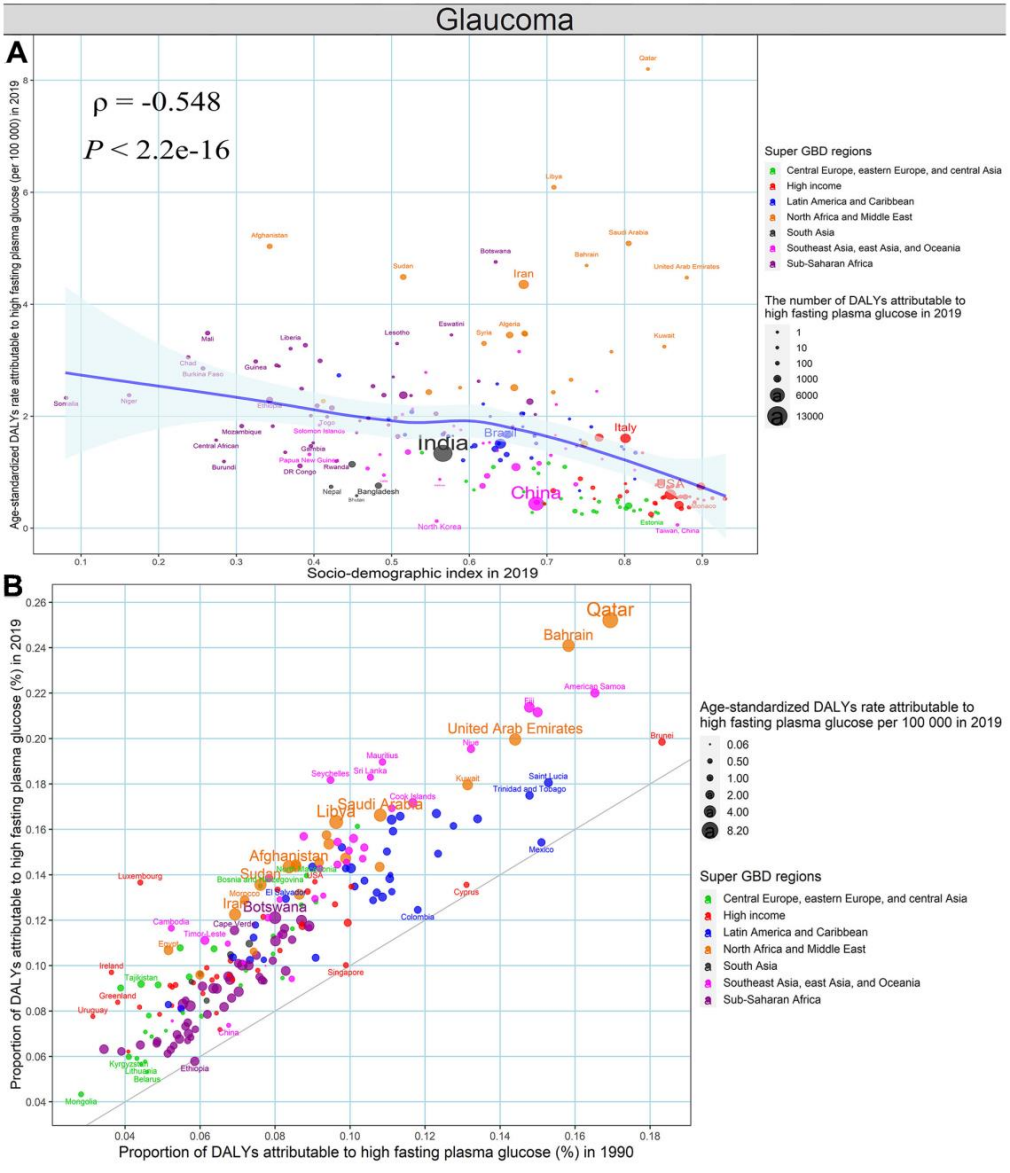
**High fasting plasma glucose contributing to glaucoma-related DALYs, both sexes, at the national level.** (**A**) The association between age-standardized DALYs rates of glaucoma attributable to high fasting plasma glucose in 2019 and SDI in 2019. (**B**) The proportion of DALYs of glaucoma attributable to high fasting plasma glucose in 1990 and 2019. DALYs, disability-adjusted life years; SDI, socio-demographic index.

**Figure 9:**
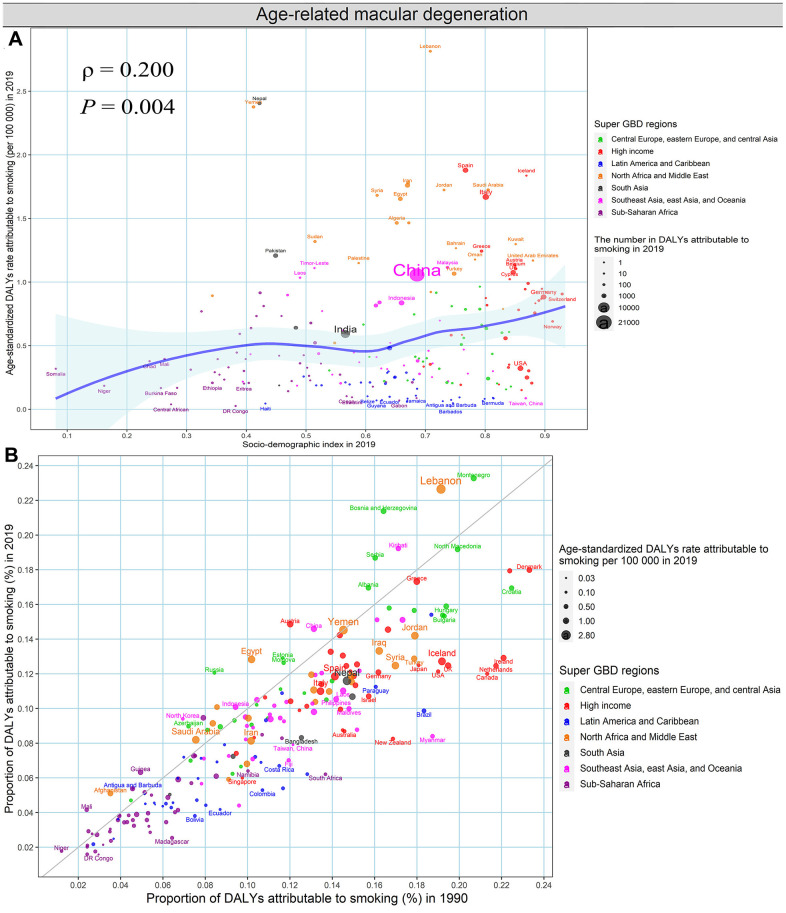
**Smoking contributing to DALYs of age-related macular degeneration, both sexes, at the national level.** (**A**) The association between age-standardized DALYs rates of age-related macular degeneration attributable to smoking in 2019 and SDI in 2019. (**B**) The proportion of DALYs of age-related macular degeneration attributable to smoking in 1990 and 2019. DALYs, disability-adjusted life years; SDI, socio-demographic index.

The age-standardized DALYs rate of glaucoma attributable to high fasting plasma glucose in 2019 was negatively associated with SDI in 2019 at the national levels (ρ = -0.548, *P* < 2.2e-16) (Figure 8). Besides, the proportion of glaucoma-related DALYs due to high fasting plasma glucose increased in almost all countries and territories between 1990 to 2019, except for Ethiopia. (Figure 8 and Supplementary Table 6). We could detect a significant association between the age-standardized DALYs rate of age-related macular degeneration attributable to smoking in 2019 and SDI in 2019 at the national levels (ρ = 0.200, *P* = 0.004) (Figure 9). The proportion of DALYs of age-related macular degeneration due to smoking decreased among most countries and territories during the monitoring period, except for 33 countries and territories, including Bosnia and Herzegovina, Russian and Lebanon, etc., (Figure 9 and Supplementary Tables 7, 8).

## DISCUSSION

We comprehensively summarized the global, regional, and national burden in prevalence and DALYs of BVL by specific eye diseases along with the temporal trend over the past three decades in 204 countries and territories. From a global perspective, the prevalence and DALYs cases of BVL were continuously increasing, with an estimated 0.71 billion people suffering from BVL in 2019, while the age-standardized rates only presented a mild downward trend during the period. However, the heterogeneous patterns in specific eye diseases, regions, sexes and age groups across the world make the prevention and healthcare of BVL complex [1, 7]. For example, the age-standardized DALYs rate caused by age-related macular degeneration increased in Sub-Saharan Africa, but the age-standardized DALYs rate caused by near vision loss increased in Eastern Europe. Therefore, understanding the exact change pattern of the BVL burden is essential for policy-makers to allocate rationally limited medical resources and formulate adapted prevention and treatment strategies.

The top three eye diseases for DALYs due to BVL remained cataract, refractive disorders, and near vision loss over the past three decades. The DALYs due to cataract tended to be more prevalent in low or low-middle SDI regions, while it is promising that the age-standardized DALYs rates due to cataract in these regions have been decreasing over time, except for Burkina Faso, Chad, Afghanistan and Guinea, etc. The identified main contribution to cataract, namely, air pollution from solid fuels and smoking, dropped shapely across most countries, because the clean fuel for cooking has been popularized gradually with economic development and environmental requirement [8–10], and the prevalence of tobacco smoking further fell in more than 125 countries since 2003 under the WHO Framework Convention on Tobacco Control (FCTC) mobilization across the world [11, 12]. However, the age-standardized DALYs rates due to household air pollution from solid fuels in low SDI and low-middle regions still exceeded 74/100 000 over the past 30 years, especially in women, which was more than 300 times the burden in the high SDI region in 2019. A recent study from India reported that the application of clean cooking fuels such as kerosene or liquefied petroleum gas didn’t significantly increase with income growth, because the housework of cooking and fuel collection remains primarily women’s responsibility and gender inequality within the family determines low household investment in clean fuel in developing areas [13]. Promoting healthy lifestyles and eliminating gender inequality can alleviate the corresponding burden. Moreover, we noted that the age-standardized DALYs rate of cataract attributed to high body-mass index and high fasting plasma glucose elevated gradually across almost all SDI regions and even surpassed the contributor of smoking and air pollution from solid fuels in the high SDI region. Despite the improvement in prevention and treatment, cataract remains a major threat to eye health and accounts for one-third blindness [7, 14].

Unlike other BVL eye diseases, the distribution in DALYs due to refractive disorders did not exhibit an obvious association with SDI levels, and the corresponding geographical variation was lowest, except for a high burden in South Asia. The age-standardized DALYs rates due to refractive disorders in almost high-burden areas have been declining, which is consistent with the previous studies [15, 16]. Although the rate of refractive disorders slightly increased with age, the number of DALYs due to refractive disorders remained relatively stable across age groups among the population under 80 years old, which indicates that eye health promotion among young people may decrease the burden [17]. No clear risk factors for refractive disorders were evaluated in the GBD study, but some studies reported that education years, unreasonable use of eyes, fine particulate matter and ozone in the environment are associated with refractive disorders [18–20].

The DALYs due to near vision loss tended to be pronounced in Sub-Sahara Africa and India. Of all the identified BVL eye diseases, only the age-standardized DALYs rate due to near vision loss in most countries and regions remained relatively stable over the last couple of decades, which means the number of near vision loss patients would still shapely increase under the population growth in Sub-Sahara Africa and India. The relatively stable rate might be related to the negligence of the control of near vision loss in public health policy, because of the safe and effective treatment with optical correction [21], which is also illustrated by the difference between prevalence and DALYs proportion. Albeit, no potential environmental risk factor was well-identified for near vision loss in the GBD study, we observed that the burden due to near vision loss in high-risk areas was highly related to the low socio-economic status, which was in agreement with the previous studies [22, 23]. Low socioeconomic status usually indicates poor awareness of vision loss, less access to vision correction, lack of ophthalmologists and optometrists, and inability to afford suitable glasses. Reasonable eye use, eye care exercises, and the availability of glasses could reduce the burden of near vision loss [24, 25].

The age-standardized DALYs rates due to glaucoma and age-related macular degeneration were significantly lower than the above-mentioned eye diseases, but they are responsible for about one-sixth of blindness [16, 21]. The geographical distributions for glaucoma and age-related macular degeneration were highly similar, and the disease burden was pronounced in low SDI areas, except the high burden of age-related macular degeneration was found in Western Europe [26–28]. The obvious decline in DALYs due to glaucoma was detected in 194 countries and territories, especially in high-burden areas, but the proportion of glaucoma-related DALYs due to high fasting plasma glucose increased in almost all countries and territories, which further emphasizes the importance of the prevention and control of high fasting plasma glucose in public policy [29, 30]. By contrast, 181 countries and territories presented a downward trend in DALYs due to age-related macular degeneration, and the contribution of smoking fell in most countries and territories, with the implementation of various tobacco control measures [12]. Some studies have indicated that the high smoking prevalence, Western diet pattern, increased high-density lipoprotein, low 25-hydroxyvitamin D, and decreased amino acids are the risk factors for age-related macular degeneration [12, 31–36], which may contribute to the high burden in high SDI areas.

Apart from glaucoma, cataract, age-related macular degeneration, refraction disorders and near vision loss, other eye diseases such as diabetic retinopathy, retinopathy of prematurity, trachoma, and onchocerciasis, were integrated into “other vision loss” in this study; these have also been recognized as causations of BVL [21, 37, 38]. In the current study, we observed that age-standardized DALYs rates due to other vision loss decreased in 193 regions, especially in North Africa and Middle East, and Southeast Asia. However, the high-burden areas, including most Western Sub-Saharan Africa countries and Afghanistan, presented a minor increased trend. Our results may indicate that the various risk factors responsible for other vision loss, such as the lack of access to water and crowded living conditions that cause trachoma [39], need to be improved, and the available treatment, such as the use of azithromycin for trachoma [40], need to be strengthened, especially in high-burden areas.

In addition, we found that the temporal trend in age-standardized DALYs rate due to BVL from 1990 to 2019, namely EAPC, was significantly negatively associated with baseline age-standardized rate. For those heavily burdened countries in 1990, the age-standardized DALYs rate of BVL was more likely to decline, which could be explained by the fact that countries with high burden are likely to take into account BVL and identified risk factors as a high priority in disease prevention and treatment schemes [41]. The annualized declined trend of age-standardized DALYs rates due to BVL was associated with the increasing SDI, except for the SDI areas exceeding 0.8 with a low burden in BVL. Possible reasons involve the highly public attention to BVL, intensive eye health education, the availability of clean cooking fuel, effective tobacco control intervention, access to visual examination, and improved vision correction and treatment [22, 42].

Some deficiencies should be taken into account in the interpretation of our results. First, although the collaborators in GBD 2019 study have done enormous work every year to assess the disease burden worldwide, bias could not be avoided in fitting the unavailable data, as described previously [2, 29]. Second, the heterogeneity in quality and quantity of data sources, diagnosis of BVL and its disease subtypes, assessment for risk factors of BVL in different regions might hamper the conclusion.

BVL is still one of the major global public health concerns. Albeit minor achievements have been attained in the age-standardized burden rate in most countries and territories, the absolute BVL burden continues increasing rapidly in the context of population growth and aging, especially in low SDI areas. We need to further strengthen tobacco control and replace solid fuels with clean energy to minimize household air pollution. Meanwhile, we should control the body-mass index and fasting plasma glucose to reduce the related BVL burden. Fortunately, WHO united with Member States and their partners have initiated and implemented several related programs, such as Vision 2020 the right to sight, Universal eye health: a global action plan 2014-2019, and World report on vision. These programs will help to reduce the BVL burden by providing guidance and technical support for comprehensive vision protection systems and promoting the available ophthalmic care. The information addressed in the current study would clarify the global disease burden of BVL by specific eye diseases and formulate more effective and targeted BVL prevention and healthcare strategies.

## MATERIALS AND METHODS

### Data source

The detailed resources search, inclusion and exclusion criteria, data processing steps and modeling methods of GBD 2019 have been delineated in previous GBD studies [2, 29], and the repeatable analytical process and statistical codes for estimating BVL burden could be obtained from the accessible supporting website (http://ghdx.healthdata.org/gbd-2019/code). Here, we briefly introduced the methods specific to the estimation of the BVL burden. Each step performed in this study to analyze and report the GBD database complied with the Guidelines for Accurate and Transparent Health Estimates Reporting (GATHER) statements [43]. The following search terms were used to collect BVL burden information on MEDLINE, Embase, WHOLIS, SciELO, Open Grey and other gray literature searches: (“glaucoma” OR “cataract” OR “macular” OR “refractive error” OR “presbyopia” OR “blindness” OR “vision, low”) AND (“prevalence” OR “incidence” OR “epidemiology”). A total of 387 original BVL-related data sources were extracted for this cause-specific assessment, covering representative population-based studies, peer-reviewed publications, grey literature and recorded surveys. All ICD-9 and ICD-10 codes pertaining to BVL (360.8-362, 362.1-363.9, 365-369.9, 377-378.9 and H25-H28.8, H31-H36.8, H40-H40.9, H42-H42.8, H46-H54.9, respectively) were claimed in the GBD estimates. The Disease Modelling Meta-Regression (DisMod-MR) 2.1 modeling was developed by the GBD Collaborators to estimate non-fatal diseases burden based on Bayesian mixed-effects meta-regression [2]. In the DisMod model, all original data from all available regions was input into a mixed-effects nonlinear model to produce a global estimation of BVL burden as well as fixed and random effects. The model outputs were delivered to the regional level as a prior to fit the regional estimations, which were further used as the prior values to fit the national estimations. The 95% uncertainty interval (UI) for all estimations was generated using the 2.5th and 97.5th centiles of 1000 random draws of the posterior distribution.

We exacted annual data on BVL burden by sex, 5-year age group and eye diseases (glaucoma, cataract, age-related macular degeneration, refraction disorders, near vision loss, and, other vision loss) in 204 countries and regions from 1990 to 2019 from the Institute for Health Metrics and Evaluation (http://ghdx.healthdata.org/gbd-results-tool). In order to delineate the disease burden of BVL across the world, the 204 available countries and territories were divided into five regions according to the corresponding SDI, a comprehensive indicator combining per capita income, years of education, and fertility, namely, low, low-middle, middle, high-middle, and high SDI regions. Besides, the world was geographically categorized into 21 GBD areas, e.g., high-income Asia Pacific, Tropical Latin America, and West Europe (Table 1), which were further simplified into seven super GBD regions.

Among 87 behavioral, environmental and occupational, and metabolic risk factors quantified in GBD 2019, the attributable risks of smoking, high body-mass index, household air pollution from solid fuels, and high fasting plasma glucose for BVL burden were well estimated, based on the established risk–outcome inclusion criterion. The comparative risk assessment framework was applied to calculate the population attributable fraction of potential risk factors for disease-specific BVL burden [29]: identify convincing risk-outcome pairs with the summary relative risk based on systematic review and meta-regression, estimate the exposure level and distribution by spatio-temporal Gaussian process regression and Bayesian mixed-effects meta-regression, define the theoretical minimum risk exposure level, and calculate the population attributable fraction and attributable burden.

### Statistical analysis

The ASPR and age-standardized DALYs rate were used to quantify the variation of BVL burden by calendar year, sex, and region, to avoid the differences in the age composition of the population, based on the WHO World Standard Population Distribution 2001. We calculated the EAPC to describe the temporal trend in various age-standardized rates of BVL burden. We performed a regression model to fit the natural logarithm of the age-standardized rate with the calendar year, namely, ln (age-standardized rate) = α + β* calendar year + ϵ, as our previous articles [44, 45]. The EAPC with its 95%CI were estimated according to the formula of 100 × (exp (β) − 1). In our study, the 95% lower boundary of EAPC greater than 0 indicated that the changing trend was increasing, whereas the 95% upper boundary of EAPC less than 0 indicated that the changing trend was decreasing. We applied the Spearman rank test to calculate the relationship correlation between the EAPCs in BVL-related burden and the baseline burden in 1990 and the SDI in 2019 at the national level. The age-standardized rate of BVL burden in 1990 could be used as a proxy for the baseline disease reservoir, and the SDI in 2019 could represent the level and availability of health care in each country. All statistical analyses in the current study were conducted using R program version 4.0.3 (https://www.R-project.org/), and the two-sided *P* value less than 0.05 was considered statistically significant, which was presented in scientific notation format when it was less than 0.001.

### Ethics approval and consent to participate

The GBD 2019 study is a publicly available database and all data were anonymous. Our study protocol was approved by the Institutional Review Boards of Qilu Hospital of Shandong University with approval number KYLL-202011(KS)-134.

### Data availability statement

All data could be extracted from the online database (http://ghdx.healthdata.org/gbd-results-tool).

## Supplementary Material

Supplementary Figures

Supplementary Tables
