# Supplementary material for: Global, regional, and national burden of blindness and vision loss due to common eye diseases along with its attributable risk factors from 1990 to 2019: a systematic analysis from the global burden of disease study 2019
Source: Aging (Albany NY). 2021 Aug 9;13(15):19614–42. doi: 10.18632/aging.203374 (PMC8386528; doi:10.18632/aging.203374)
Supplement: Supplementary Figures [file aging-13-203374-s001.pdf]

SUPPLEMENTARY FIGURES

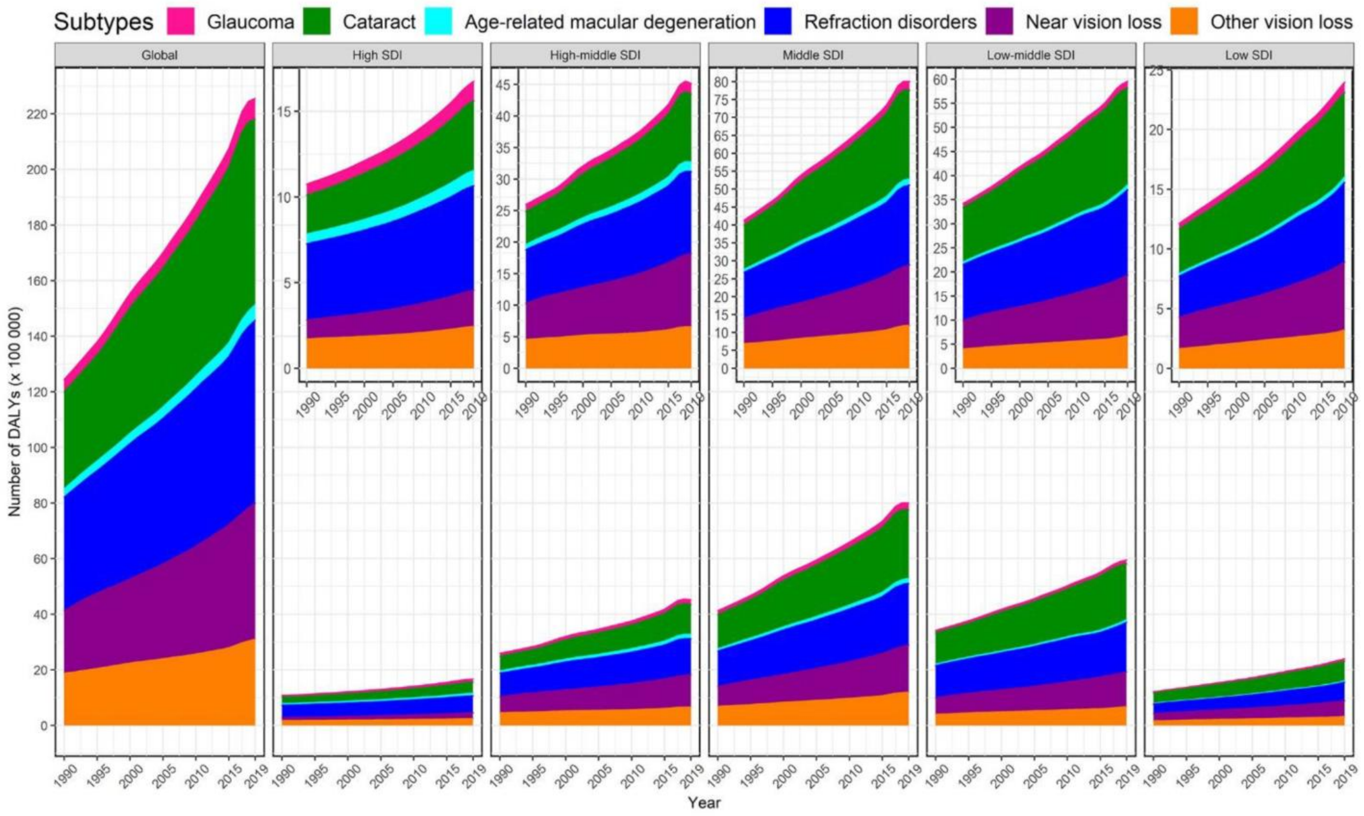

Supplementary Figure 1. The DALYs number of BVL by different eye diseases, by SDI regions, from 1990 to 2019. The data from five SDI regions are further enlarged in its top-right panel. DALYs, disability-adjusted life years; BVL, blindness and vision loss; SDI, socio-demographic index.

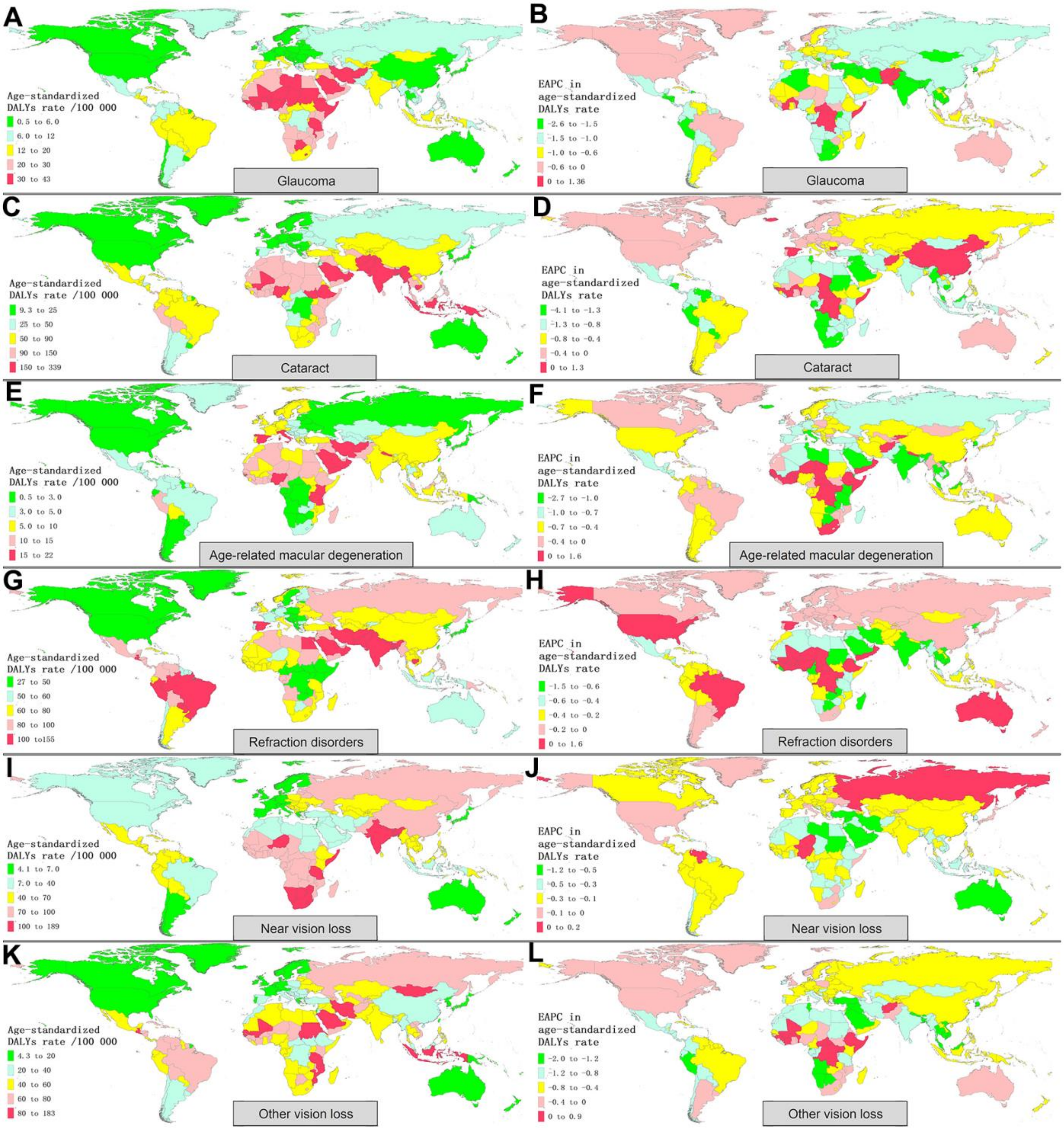

Supplementary Figure 2. The age-standardized DALYs rate and the EAPC in age-standardized DALYs rate of BVL by six eye diseases for both sexes in 204 countries and territories. (**A**, **B**) glaucoma; (**C**, **D**) cataract; (**E**, **F**) age-related macular degeneration; (**G**, **H**) refraction disorders; (**I**, **J**) near vision loss; (**K**, **L**) other vision loss. DALYs, disability-adjusted life years; EAPC, estimated annual percentage change.

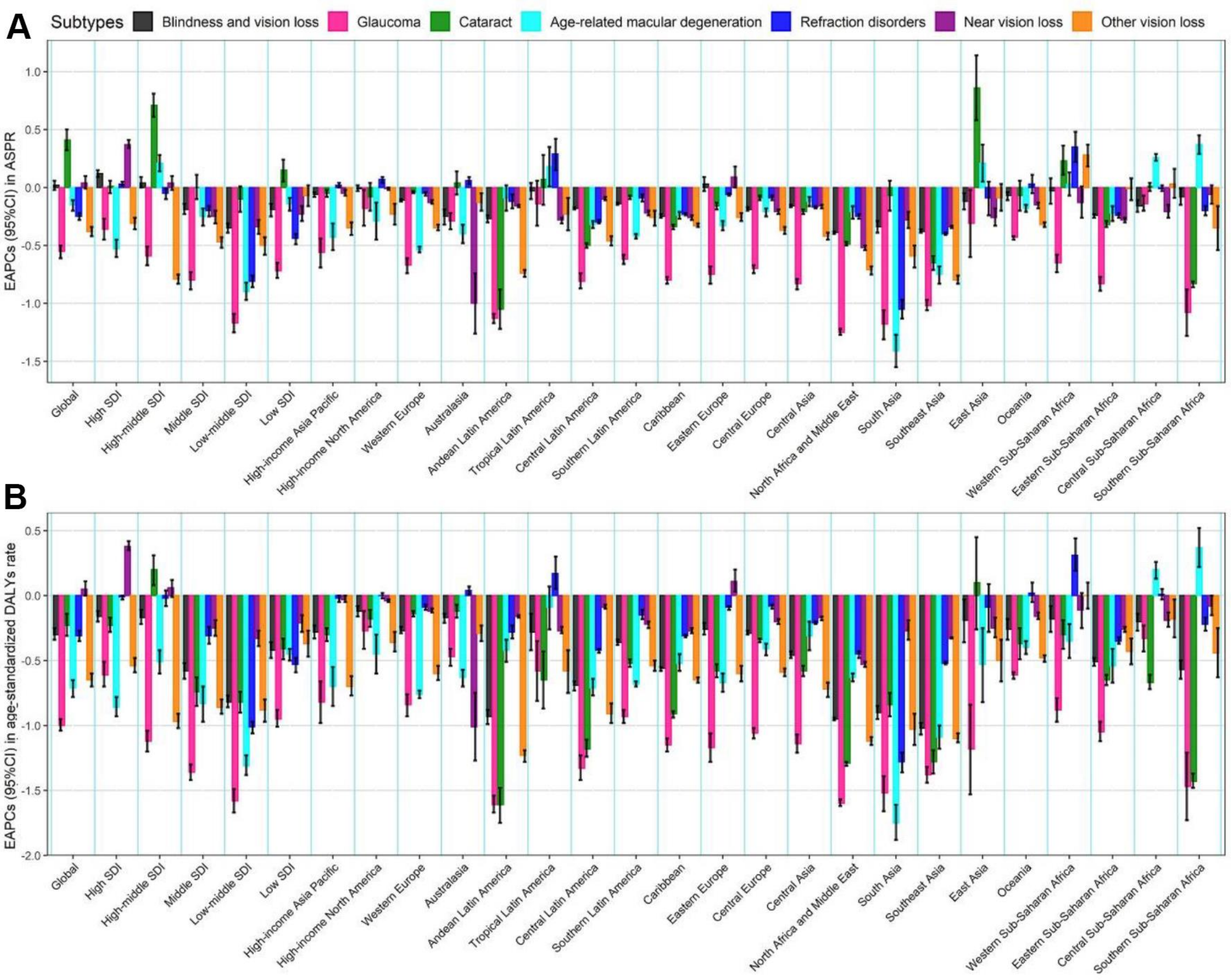

Supplementary Figure 3. The EAPCs of BVL burden from 1990 to 2019, both sexes, by region and eye diseases. (**A**) The EAPCs in ASPR of BVL; (**B**) The EAPCs in age-standardized DALYs rate of BVL. EAPC, estimated annual percentage change; BVL, blindness and vision loss; ASPR, age-standardized prevalence rate; DALYs, disability-adjusted life years.

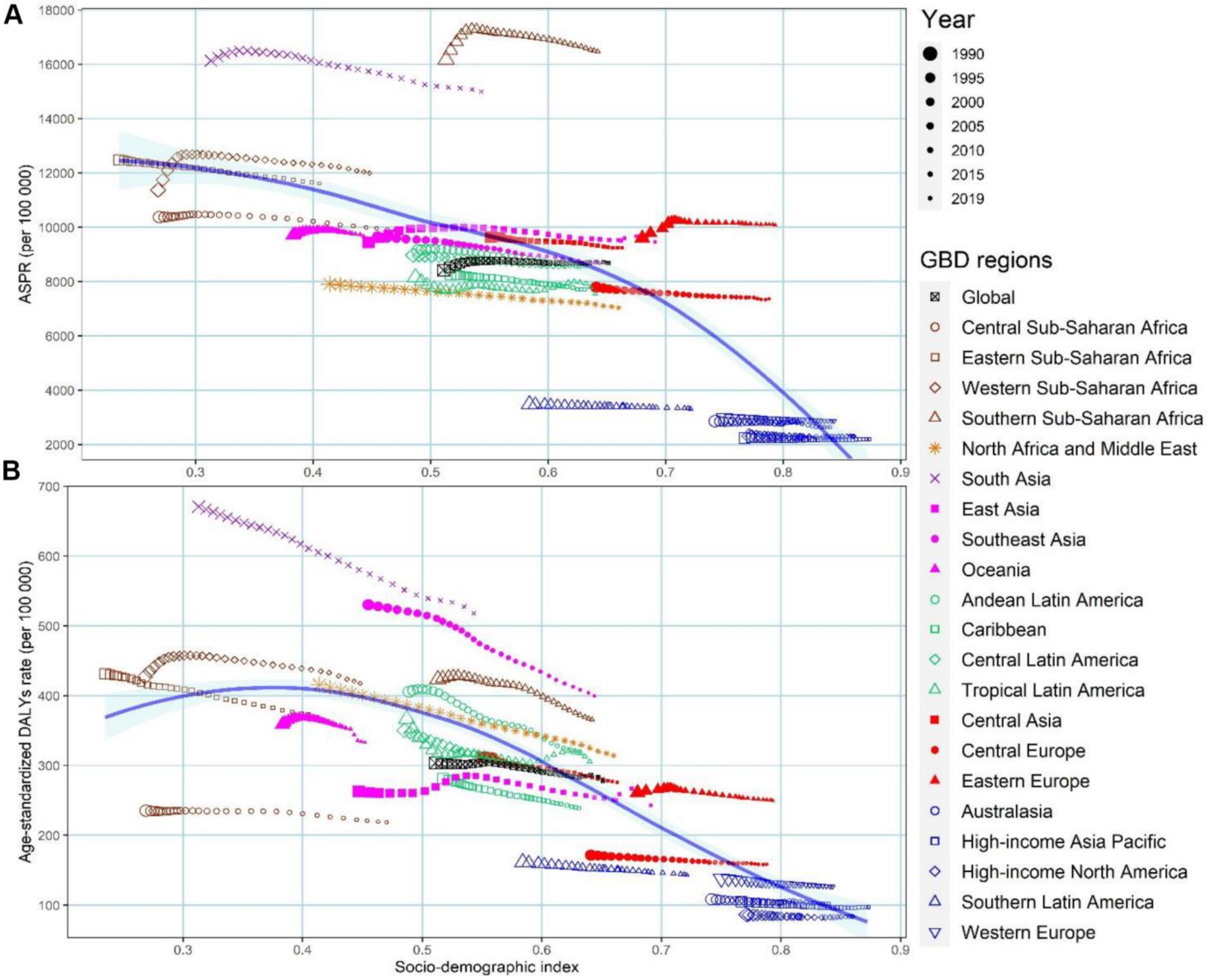

Supplementary Figure 4. The changing trend in age-standardized burden rate of overall BVL across 21 GBD regions with SDI, both sexes, from 1990 to 2019. (**A**) ASPR; (**B**) age-standardized DALYs rate. BVL, blindness and vision loss; GBD, global burden of disease; SDI, socio-demographic index; ASPR, age-standardized prevalence rate; DALYs, disability-adjusted life years.
