# Supplementary material for: Global, regional, and national burden of blindness and vision loss due to common eye diseases along with its attributable risk factors from 1990 to 2019: a systematic analysis from the global burden of disease study 2019
Source: Aging (Albany NY). 2021 Aug 9;13(15):19614–42. doi: 10.18632/aging.203374 (PMC8386528; doi:10.18632/aging.203374)
Supplement: Supplementary Tables [file aging-13-203374-s002.pdf]

SUPPLEMENTARY TABLES

Supplementary Table 1. Prevalence cases and age-standardized prevalence rate per 100 people for all blindness and vision loss in 1990 and 2019, and its estimated annual percentage change from 1990 to 2019.

Characteristics	1990		2019		EAPC in ASPR, 1990-2019
	Prevalence cases No.×10^6 (95% UI)	ASPR per 100 No. (95% UI)	Prevalence cases No.×10^6 (95% UI)	ASPR per 100 No. (95% UI)	No. (95% CI)
**Overall**	353.2 (298.7, 414.0)	8.43 (7.07, 9.86)	713.9 (593.2, 841.1)	8.69 (7.27, 10.22)	0.02 (-0.02, 0.06)
**Sex**					
Males	162.2 (136.6, 191.2)	8.18 (6.88, 9.61)	323.6 (268.9, 383.8)	8.25 (6.91, 9.73)	-0.06 (-0.10, -0.02)*
Females	190.9 (161.4, 222.6)	8.67 (7.29, 10.11)	390.4 (325.0, 458.9)	9.10 (7.61, 10.68)	0.09 (0.05, 0.12)*
**SDI**					
High SDI	28.30 (24.92, 32.03)	2.97 (2.63, 3.34)	47.11 (41.14, 53.83)	3.08 (2.72, 3.48)	0.12 (0.09, 0.15)*
High-middle SDI	85.69 (70.93, 102.1)	8.00 (6.67, 9.50)	162.5 (133.2, 194.6)	8.31 (6.90, 9.88)	0.04 (0.00, 0.09)*
Middle SDI	111.8 (93.78, 132.0)	10.12 (8.44, 11.92)	247.0 (204.3, 293.6)	9.82 (8.22, 11.61)	-0.19 (-0.23, -0.14)*
Low-middle SDI	90.84 (76.88, 106.5)	13.57 (11.47, 15.79)	179.8 (149.2, 213.2)	12.5 (10.45, 14.73)	-0.35 (-0.39, -0.31)*
Low SDI	36.40 (30.48, 42.71)	13.63 (11.43, 15.95)	77.22 (65.04, 90.76)	13.13 (10.98, 15.37)	-0.19 (-0.25, -0.14)*
**GBD region**					
High-income Asia Pacific	4.14 (3.71, 4.59)	2.25 (2.03, 2.48)	7.26 (6.47, 8.11)	2.21 (1.99, 2.44)	-0.06 (-0.08, -0.04)*
High-income North America	7.54 (6.65, 8.54)	2.32 (2.05, 2.62)	11.91 (10.39, 13.61)	2.31 (2.04, 2.61)	-0.01 (-0.03, 0.02)
Western Europe	14.40 (13.03, 15.89)	2.98 (2.70, 3.26)	19.60 (17.66, 21.68)	2.87 (2.59, 3.15)	-0.11 (-0.12, -0.10)*
Australasia	0.62 (0.57, 0.68)	2.86 (2.61, 3.12)	1.04 (0.92, 1.17)	2.64 (2.35, 2.94)	-0.25 (-0.32, -0.19)*
Tropical Latin America	8.68 (7.66, 9.77)	8.16 (7.15, 9.32)	17.90 (15.61, 20.43)	7.55 (6.60, 8.62)	-0.03 (-0.10, 0.04)
Andean Latin America	2.17 (1.90, 2.46)	9.15 (7.95, 10.50)	4.93 (4.25, 5.66)	8.56 (7.38, 9.87)	-0.27 (-0.30, -0.24)*
Central Latin America	8.61 (7.43, 9.96)	8.98 (7.62, 10.56)	20.40 (17.20, 24.06)	8.55 (7.22, 10.11)	-0.18 (-0.19, -0.17)*
*Southern Latin America	1.63 (1.48, 1.80)	3.48 (3.16, 3.82)	2.52 (2.28, 2.78)	3.32 (3.01, 3.65)	-0.14 (-0.15, -0.13)*
Caribbean	2.27 (1.91, 2.69)	8.26 (6.89, 9.86)	3.95 (3.27, 4.72)	7.72 (6.43, 9.21)	-0.25 (-0.26, -0.23)*
Eastern Europe	25.92 (21.28, 31.08)	9.61 (7.95, 11.45)	33.05 (26.85, 40.07)	10.1 (8.27, 12.13)	0.03 (-0.03, 0.09)
Central Europe	11.17 (8.88, 13.89)	7.79 (6.25, 9.64)	14.84 (11.75, 18.68)	7.37 (5.92, 9.13)	-0.19 (-0.20, -0.17)*
Central Asia	4.65 (3.87, 5.51)	9.64 (7.97, 11.45)	6.93 (5.71, 8.3)	9.25 (7.67, 11.11)	-0.16 (-0.17, -0.15)*
North Africa and Middle East	15.98 (14.19, 17.84)	7.92 (6.99, 8.95)	32.49 (28.62, 36.58)	7.04 (6.19, 8.00)	-0.39 (-0.40, -0.38)*
South Asia	104.7 (89.03, 122.1)	16.14 (13.75, 18.72)	224.1 (187.5, 265.6)	15.00 (12.58, 17.61)	-0.34 (-0.39, -0.29)*
Southeast Asia	26.88 (23.47, 30.48)	9.64 (8.41, 10.96)	52.77 (45.5, 60.45)	8.67 (7.54, 9.95)	-0.38 (-0.39, -0.36)*
East Asia	84.75 (67.36, 104.5)	9.44 (7.56, 11.57)	195.9 (155.6, 240.3)	9.47 (7.66, 11.49)	-0.12 (-0.19, -0.05)*
Oceania	0.32 (0.27, 0.37)	9.72 (8.28, 11.36)	0.73 (0.62, 0.85)	9.55 (8.14, 11.15)	-0.07 (-0.11, -0.04)*
Western Sub-Saharan Africa	10.99 (9.06, 13.19)	11.38 (9.35, 13.64)	26.13 (21.62, 31.21)	12.00 (9.92, 14.34)	-0.03 (-0.14, 0.08)*
Eastern Sub-Saharan Africa	10.35 (8.45, 12.3)	12.49 (10.22, 14.89)	21.47 (17.69, 25.53)	11.62 (9.57, 13.76)	-0.24 (-0.26, -0.23)*
Central Sub-Saharan Africa	2.55 (2.01, 3.17)	10.38 (8.13, 12.84)	5.80 (4.60, 7.24)	9.89 (7.85, 12.30)	-0.16 (-0.21, -0.11)*
Southern Sub-Saharan Africa	4.88 (3.88, 5.96)	16.18 (12.77, 19.68)	10.20 (8.00, 12.49)	16.48 (13.05, 20.19)	-0.08 (-0.15, -0.01)*
**Specific subtypes**					
Glaucoma	3.88 (3.30, 4.54)	0.11 (0.09, 0.13)	7.47 (6.35, 8.77)	0.09 (0.08, 0.11)	-0.55 (-0.61, -0.50)*
Cataract	42.34 (37.73, 47.62)	1.15 (1.03, 1.29)	97.02 (85.37, 109.7)	1.21 (1.07, 1.36)	0.41 (0.32, 0.50)*
Age-related macular degeneration	3.58 (3.03, 4.19)	0.10 (0.08, 0.11)	7.79 (6.53, 9.16)	0.10 (0.08, 0.11)	-0.15 (-0.20, -0.11)*
Refraction disorders	97.61 (87.51, 108.5)	2.08 (1.87, 2.31)	157.4 (140.9, 174.9)	1.96 (1.75, 2.18)	-0.25 (-0.28, -0.22)*
Near vision loss	227.2 (164.2, 298.4)	5.61 (4.08, 7.34)	493.2 (358.9, 645.6)	5.94 (4.34, 7.77)	0.04 (-0.01, 0.10)
Other vision loss	21.10 (19.06, 23.56)	0.52 (0.47, 0.58)	38.40 (34.35, 43.08)	0.47 (0.43, 0.53)	-0.38 (-0.42, -0.34)*

No., number; ASPR, age-standardized prevalence rate; UI, uncertainty interval; EAPC, estimated annual percentage change; CI, confidential interval; SDI, Socio-demographic Index; GBD, global burden of disease.
* *P* value<0.05.

Supplementary Table 2. Countries/territories with ASPR per 100 greater than 11 in 2019.

Number	Location	ASPR per 100 No. (95% UI)
1	Nepal	21.27 (18.16, 24.24)
2	Tanzania	18.01 (14.90, 21.20)
3	Zimbabwe	16.97 (13.31, 20.93)
4	Lesotho	16.75 (13.16, 20.80)
5	South Africa	16.45 (13.06, 20.13)
6	Eswatini	16.27 (12.58, 20.19)
7	Namibia	15.88 (12.24, 19.87)
8	Botswana	15.78 (12.25, 19.56)
9	Niger	15.41 (13.08, 17.92)
10	India	15.29 (12.79, 18.05)
11	Bangladesh	13.78 (11.39, 16.55)
12	Nigeria	12.87 (10.63, 15.31)
13	Pakistan	12.42 (10.87, 14.02)
14	Bhutan	12.39 (9.74, 15.45)
15	Somalia	12.39 (9.57, 15.43)
16	Mozambique	11.96 (9.62, 14.61)
17	Mali	11.95 (9.82, 14.52)
18	Malawi	11.81 (9.49, 14.41)
19	South Sudan	11.59 (9.33, 14.18)
20	Burkina Faso	11.57 (9.36, 14.01)
21	Eritrea	11.54 (9.29, 13.97)
22	Guinea	11.45 (9.31, 14.02)
23	Moldova	11.40 (9.29, 13.74)
24	Guinea-Bissau	11.39 (9.31, 13.83)
25	Chad	11.38 (9.23, 13.87)
26	Madagascar	11.14 (8.80, 13.74)
27	Benin	11.07 (8.98, 13.36)
28	Comoros	11.07 (8.81, 13.53)
29	Gambia	11.05 (8.99, 13.56)

ASPR, age-standardized prevalence rate; No., number; UI, uncertainty interval.

Supplementary Table 3. Countries/territories with age-standardized DALYs rate per 1000 greater than 3.8 in 2019.

Number	Location	Age-standardized DALYs rate per 1000 No. (95% UI)
1	Indonesia	5.59 (4.04, 7.48)
2	Pakistan	5.39 (3.85, 7.30)
3	India	5.25 (3.66, 7.27)
4	Mali	5.13 (3.62, 7.02)
5	Timor-Leste	4.99 (3.52, 6.81)
6	Nepal	4.78 (3.14, 7.20)
7	Tanzania	4.75 (3.21, 6.73)
8	Afghanistan	4.70 (3.33, 6.33)
9	Nigeria	4.68 (3.30, 6.47)
10	Saudi Arabia	4.65 (3.32, 6.22)
11	Oman	4.55 (3.26, 6.15)
12	Bangladesh	4.48 (3.06, 6.33)
13	Cambodia	4.47 (3.17, 6.12)
14	Niger	4.34 (2.99, 6.05)
15	Lesotho	4.23 (2.88, 6.11)
16	Myanmar	4.21 (2.96, 5.75)
17	Ethiopia	4.21 (3.03, 5.69)
18	Zimbabwe	4.20 (2.87, 6.20)
19	Guinea-Bissau	4.18 (2.94, 5.82)
20	Guinea	4.04 (2.80, 5.60)
21	Senegal	3.96 (2.79, 5.47)
22	Burkina Faso	3.94 (2.76, 5.45)
23	Eswatini	3.91 (2.66, 5.76)
24	South Sudan	3.87 (2.70, 5.35)
25	Eritrea	3.86 (2.68, 5.35)
26	Botswana	3.83 (2.63, 5.65)
27	Mozambique	3.81 (2.66, 5.28)
28	Guatemala	3.80 (2.65, 5.21)

DALYs, disability-adjusted life years; No., number; UI, uncertainty interval.

Supplementary Table 4. Top 20 countries/territories with the most rapid decrease in age-standardized DALYs rate from 1990 to 2019.

Number	Location	Age-standardized DALYs rate per 1000 in 1990 No. (95% UI)	Age-standardized DALYs rate per 1000 in 2019 No. (95% UI)	EAPC in age-standardized DALYs rate No. (95% CI)
1	Equatorial Guinea	6.25 (4.44, 8.41)	3.34 (2.33, 4.63)	-2.45 (-2.59, -2.31)
2	Saudi Arabia	8.04 (5.73, 10.77)	4.65 (3.32, 6.22)	-1.85 (-1.91, -1.78)
3	Cambodia	6.45 (4.54, 8.72)	4.47 (3.17, 6.12)	-1.44 (-1.52, -1.36)
4	Qatar	4.10 (2.92, 5.49)	2.87 (2.02, 3.94)	-1.29 (-1.35, -1.23)
5	Tunisia	3.97 (2.81, 5.39)	2.79 (1.97, 3.78)	-1.26 (-1.33, -1.20)
6	Malaysia	4.35 (3.07, 5.90)	3.14 (2.20, 4.35)	-1.14 (-1.17, -1.10)
7	Vietnam	4.18 (2.96, 5.71)	3.09 (2.15, 4.34)	-1.11 (-1.16, -1.07)
8	Bhutan	3.91 (2.68, 5.69)	2.90 (1.91, 4.31)	-1.10 (-1.15, -1.05)
9	Thailand	3.77 (2.59, 5.26)	2.83 (1.92, 4.10)	-1.10 (-1.16, -1.05)
10	Peru	4.56 (3.24, 6.19)	3.54 (2.47, 4.91)	-1.07 (-1.17, -0.98)
11	Lebanon	4.15 (2.94, 5.61)	3.08 (2.16, 4.19)	-1.06 (-1.09, -1.03)
12	Syria	4.45 (3.15, 5.95)	3.31 (2.34, 4.51)	-1.05 (-1.12, -0.98)
13	Bahrain	3.84 (2.74, 5.17)	2.87 (2.01, 3.92)	-1.04 (-1.10, -0.99)
14	Myanmar	5.40 (3.87, 7.34)	4.21 (2.96, 5.75)	-1.01 (-1.13, -0.88)
15	Sudan	5.10 (3.66, 6.87)	3.73 (2.67, 5.03)	-1.01 (-1.08, -0.95)
16	Iraq	4.16 (2.96, 5.58)	3.16 (2.24, 4.30)	-1.01 (-1.06, -0.97)
17	India	6.95 (4.97, 9.48)	5.25 (3.66, 7.27)	-0.98 (-1.02, -0.93)
18	Botswana	5.06 (3.47, 7.03)	3.83 (2.63, 5.65)	-0.97 (-0.99, -0.94)
19	Palestine	4.07 (2.91, 5.52)	2.99 (2.11, 4.10)	-0.97 (-1.04, -0.90)
20	Jordan	3.11 (2.21, 4.23)	2.41 (1.68, 3.34)	-0.97 (-1.00, -0.93)

DALYs, disability-adjusted life years; No., number; CI, confidence interval.

Supplementary Table 5. Countries/territories with the increased age-standardized DALYs rate from 1990 to 2019.

Number	Location	Age-standardized DALYs rate per 1000 in 1990 No. (95% UI)	Age-standardized DALYs rate per 1000 in 2019 No. (95% UI)	EAPC in age-standardized DALYs rate No. (95% CI)
1	Burkina Faso	2.91 (2.00, 4.19)	3.94 (2.76, 5.45)	0.63 (0.35, 0.91)
2	Cote d'Ivoire	2.43 (1.65, 3.57)	3.74 (2.60, 5.21)	0.54 (0.06, 1.02)
3	Benin	3.04 (2.09, 4.40)	3.66 (2.52, 5.10)	0.52 (0.37, 0.67)
4	Central African Republic	2.20 (1.45, 3.34)	2.31 (1.53, 3.39)	0.22 (0.19, 0.26)
5	Chad	3.69 (2.56, 5.18)	3.75 (2.60, 5.18)	0.10 (0.01, 0.20)
6	Somalia	3.54 (2.46, 5.01)	3.45 (2.38, 4.87)	0.09 (0.03, 0.15)

DALYs, disability-adjusted life years; No., number; CI, confidence interval.

Supplementary Table 6. Top 20 countries/territories where the proportion of glaucoma-related DALYs attributable to high fasting plasma glucose increased between 1990 to 2019.

Number	Location	Proportion in 1990	Proportion in 2019	Proportion change
1	Luxembourg	4.40%	13.66%	9.26%
2	Seychelles	9.48%	18.17%	8.69%
3	Qatar	16.94%	25.21%	8.28%
4	Bahrain	15.83%	24.09%	8.26%
5	Mauritius	10.86%	18.97%	8.10%
6	Sri Lanka	10.54%	18.30%	7.76%
7	Micronesia	8.76%	15.69%	6.93%
8	Libya	9.63%	16.33%	6.70%
9	Fiji	14.78%	21.38%	6.60%
10	Cambodia	5.23%	11.65%	6.42%
11	Palestine	9.37%	15.75%	6.38%
12	Niue	13.22%	19.54%	6.32%
13	Marshall Islands	15.00%	21.16%	6.16%
14	Ireland	3.63%	9.70%	6.07%
15	Solomon Islands	7.84%	13.86%	6.02%
16	Afghanistan	8.37%	14.36%	5.98%
17	Sudan	7.61%	13.56%	5.95%
18	Czech	10.20%	16.13%	5.93%
19	Tunisia	9.44%	15.36%	5.92%
20	Bosnia and Herzegovina	7.61%	13.51%	5.90%

DALYs, disability-adjusted life years.

Supplementary Table 7. Countries/regions where the proportion of DALYs in age-related macular degeneration attributable to smoking increased between 1990 to 2019.

Number	Location	Proportion in 1990	Proportion in 2019	Proportion change
1	Bosnia and Herzegovina	16.42%	21.38%	4.96%
2	Russian	8.43%	12.05%	3.62%
3	Lebanon	19.13%	22.65%	3.52%
4	Austria	12.01%	14.86%	2.85%
5	Serbia	16.03%	18.69%	2.66%
6	Egypt	10.18%	12.83%	2.64%
7	Montenegro	20.68%	23.28%	2.60%
8	North Korea	6.94%	9.40%	2.47%
9	Kiribati	17.12%	19.23%	2.11%
10	Azerbaijan	7.21%	8.97%	1.77%
11	Mali	2.39%	4.16%	1.76%
12	Afghanistan	3.52%	5.11%	1.59%
13	Rwanda	7.91%	9.46%	1.55%
14	Qatar	8.55%	10.08%	1.53%
15	China	13.13%	14.59%	1.46%
16	Guinea	4.94%	6.33%	1.39%
17	Albania	15.70%	16.96%	1.26%
18	Estonia	11.66%	12.91%	1.25%
19	Moldova	11.70%	12.63%	0.94%
20	Antigua and Barbuda	4.58%	5.40%	0.82%
21	Cote d'Ivoire	4.56%	5.38%	0.82%
22	United Arab Emirates	8.35%	9.14%	0.79%
23	Georgia	8.10%	8.77%	0.67%
24	Indonesia	9.43%	10.09%	0.66%
25	Saudi Arabia	7.55%	8.20%	0.65%
26	Niger	1.21%	1.78%	0.57%
27	Sao Tome and Principe	2.48%	2.93%	0.45%
28	Kyrgyzstan	8.71%	8.95%	0.24%
29	Uzbekistan	4.48%	4.71%	0.22%
30	Bermuda	6.98%	7.19%	0.21%
31	Gabon	2.65%	2.77%	0.12%
32	Vietnam	10.44%	10.49%	0.05%
33	Malawi	5.14%	5.17%	0.02%

DALYs, disability-adjusted life years.

Supplementary Table 8. Top 20 countries/regions where the proportion of DALYs in age-related macular degeneration attributable to smoking decreased between 1990 to 2019.

Number	Location	Proportion in 1990	Proportion in 2019	Proportion change
1	Myanmar	18.73%	8.40%	-10.33%
2	Canada	21.32%	12.00%	-9.32%
3	Netherlands	21.73%	12.46%	-9.27%
4	Ireland	22.09%	12.92%	-9.17%
5	New Zealand	16.86%	8.25%	-8.61%
6	Brazil	18.33%	9.86%	-8.47%
7	South Africa	13.68%	6.21%	-7.47%
8	United Kingdom	19.47%	12.48%	-7.00%
9	USA	19.00%	12.14%	-6.86%
10	Venezuela	12.82%	6.21%	-6.60%
11	Iceland	19.18%	12.71%	-6.47%
12	Thailand	15.17%	8.78%	-6.39%
13	Mexico	11.67%	5.40%	-6.27%
14	Australia	14.57%	8.69%	-5.88%
15	Brunei Darussalam	14.49%	8.75%	-5.74%
16	Japan	18.08%	12.50%	-5.58%
17	Croatia	22.47%	16.95%	-5.52%
18	Colombia	10.70%	5.29%	-5.41%
19	Denmark	23.31%	17.99%	-5.32%
20	Sri Lanka	9.59%	4.41%	-5.18%

DALYs, disability-adjusted life years.
